# People with chronic low back pain display spatial alterations in high-density surface EMG-torque oscillations

**DOI:** 10.1038/s41598-022-19516-7

**Published:** 2022-09-07

**Authors:** Michail Arvanitidis, David Jiménez-Grande, Nadège Haouidji-Javaux, Deborah Falla, Eduardo Martinez-Valdes

**Affiliations:** Centre of Precision Rehabilitation for Spinal Pain (CPR Spine), School of Sport, Exercise and Rehabilitation Sciences, College of Life and Environmental Sciences, University of Birmingham, Birmingham, UK

**Keywords:** Neurophysiology, Musculoskeletal system

## Abstract

We quantified the relationship between spatial oscillations in surface electromyographic (sEMG) activity and trunk-extension torque in individuals with and without chronic low back pain (CLBP), during two submaximal isometric lumbar extension tasks at 20% and 50% of their maximal voluntary torque. High-density sEMG (HDsEMG) signals were recorded from the lumbar erector spinae (ES) with a 64-electrode grid, and torque signals were recorded with an isokinetic dynamometer. Coherence and cross-correlation analyses were applied between the filtered interference HDsEMG and torque signals for each submaximal contraction. Principal component analysis was used to reduce dimensionality of HDsEMG data and improve the HDsEMG-based torque estimation. sEMG-torque coherence was quantified in the δ(0–5 Hz) frequency bandwidth. Regional differences in sEMG-torque coherence were also evaluated by creating topographical coherence maps. sEMG-torque coherence in the δ band and sEMG-torque cross-correlation increased with the increase in torque in the controls but not in the CLBP group (*p* = 0.018, *p* = 0.030 respectively). As torque increased, the CLBP group increased sEMG-torque coherence in more cranial ES regions, while the opposite was observed for the controls (*p* = 0.043). Individuals with CLBP show reductions in sEMG-torque relationships possibly due to the use of compensatory strategies and regional adjustments of ES-sEMG oscillatory activity.

## Introduction

When executing a steady submaximal isometric contraction, the applied force or torque is not perfectly accurate but rather, small oscillations occur around the target torque value^1^. The neural origin of torque fluctuations has been a topic of interest for many years^1^. Previous studies have proposed that the variability in the times that motoneurons discharge action potentials could be a potential determinant for the accurate control of muscle force^2^. This variability could be determined by independent and common synaptic inputs received by motor units and/or by the nonlinear input–output properties of individual motoneurons^1^. However, recent studies have shown that the neural drive to the muscle (i.e., the neural activation signal that the muscles receive from the pool of innervating motoneurons) in low frequencies (< 10 Hz) is mainly relevant for torque generation as it reflects the common synaptic input received by the motor unit pool^3–5^. This happens because a group of activated motoneurons can act as a very selective filter, which eliminates all the independent components, partly linearizes the input–output motoneuron behavior and amplifies the common component^4^. Thus, the control of muscle force depends mainly on the amplitude of the low-frequency oscillations that reflect shared (common) synaptic input^1^.

Smooth force production depends highly on our proprioceptive senses of effort and force, with the first being generated centrally (via corollary discharges of the descending motor command), and the latter derived from peripheral sensory receptors (i.e., muscle spindles and tendon organs; reafference activity)^6^. These central and peripheral signals are sent to the somatosensory cortex via spinal, subcortical and cortical neural pathways, where they are integrated along with other somatosensory, visual, and vestibular information, resulting in the perception of applied forces^6,7^. A descending motor command is then sent to the tissues involved, resulting in an appropriate action^8^. The proprioceptive system ensures smooth force production through this interactive process, that requires constant monitoring and modulation of ascending and descending information based on the proprioceptive input, motor output, perception, and the resulting action^8^. Therefore, any perturbation to these systems, can affect the control of muscle force.

Pain can affect the ability to control muscle force, possibly due to altered afferent input from the painful area and/or neuroplastic changes in the central nervous system (e.g., changes in the organization of the sensory and motor cortices)^8,9^. For example, studies have shown that people with subacromial impingement syndrome^10^, knee osteoarthritis^11^, neck pain^12^ and chronic low back pain (CLBP)^13–15^ display reduced force steadiness. Impaired lumbar sensorimotor control, could possibly lead to suboptimal tissue loading (i.e., a disturbed body image could lead to unexpected high forces in suboptimal positions during movement, resulting in abnormal mechanical tissue loading, and ultimately stimulation of nociceptors and/or tissue strain) which can induce further pain/injury and/or perpetuation of symptoms^9^. However, the neuromuscular mechanisms responsible for poorer muscle force control when people have pain have not been fully established.

Many studies have attempted to assess the neural origin of fluctuations in muscle force by assessing surface sEMG and torque/force relationships. For instance, Yoshitake and Shinohara, (2013) previously demonstrated that the temporal characteristics of force fluctuations at low frequencies (< 5 Hz) are not only correlated with the motor unit discharge times (common drive), but also with the low frequency component of the rectified interference sEMG^16^. Taking this a step further, Moon et al., (2014) showed that the low frequency component of the rectified interference sEMG is also coherent with the oscillations in force at low frequencies which contributes to force variability^17^. Nevertheless, conventional sEMG recordings present numerous limitations since factors such as cross-talk, amplitude cancellation, electrode position and volume conductor effects (among others) can affect the relationship between sEMG and torque^18,19^. Hence, it is not surprising that only weak correlations have been observed between force and sEMG^5^.

Recent studies have shown that high-density electromyography (HDsEMG) can improve our understanding of the mechanisms responsible for the control of muscle force with HDsEMG motor unit decomposition. Indeed, HDsEMG recordings allow the identification of a large number of motor units and their discharge characteristics enable an accurate prediction of the resultant torque^5^. Nevertheless, the applicability of HDsEMG decomposition is limited to certain experimental conditions and can be only achieved for certain muscles^20^. For example, the identification of motor units from trunk muscles [i.e., erector spinae (ES)] is not currently possible with HDsEMG. Although intramuscular recordings could be potentially used to assess motor unit firing patterns from trunk muscles, their selectivity does not allow identification of a sample of motor units that is large enough to obtain an accurate representation of the exerted torque. One alternative to overcome these issues is taking advantage of HDsEMG's high spatial sampling resolution. Staudenmann et al.^21^ previously showed that HDsEMG can improve sEMG-based force estimations by 25%. This improved estimation is enabled by employing dimensionality reduction techniques such as Principal Component Analysis (PCA) which reflects the common variability between the multiple sEMG signals recorded with HDsEMG, providing a unique signal which can explain most of the variance in exerted torque^22^. In addition to PCA, HDsEMG has the possibility, not yet exploited, of generating a topographical map assessing relationships between torque and sEMG from the regions covered by the electrode grid. This would allow to determine whether certain muscle regions contribute more to the exerted torque than others. In the case of CLBP, several studies have revealed variations in lumbar ES amplitude distribution^23–27^, and in particular, studies have shown that people with CLBP often display less activity in the more caudal regions of the lumbar ES during static-fatiguing and dynamic lumbar extension tasks^23,24,26,27^. It is possible that regional variations in sEMG-torque relationships are responsible for poorer control of force during visuomotor torque-matching tasks, yet this has never been examined.

The aims of this study are to (1) quantify the relationship between HDsEMG oscillations and isometric trunk-extension torque oscillations in both time (i.e., cross-correlation) and frequency domains (i.e., coherence) and (2) examine and compare regional differences in sEMG-torque coherence of the ES muscle in individuals with CLBP and asymptomatic controls. It was hypothesized that individuals with CLBP will show a weaker correlation between HDsEMG and torque and a more focalized distribution of HDsEMG-torque coherence of the lumbar ES muscle.

## Methods

### Design and setting

This case–control, cross-sectional study was approved by the Ethics Committee of the University of Birmingham (approval number: ERN 19–1148) and work is reported in accordance with the STROBE guidelines^28^**.** All experimental procedures were conducted according to the Declaration of Helsinki. Data collection was performed between April 2019 and January 2020 at a laboratory within the Centre of Precision Rehabilitation for Spinal Pain (CPR Spine), University of Birmingham, UK. All participants attended one laboratory session and their written informed consent was obtained prior to their participation. All participants were encouraged to address any concerns or questions related to the study before signing the informed consent.

### Participants

Fifteen individuals with CLBP (seven males, eight females) and 15 asymptomatic controls (eight males, seven females) from the University of Birmingham student and staff community were recruited for this study, by means of information leaflets posted around the University of Birmingham and posts on social media. The sample size was calculated based on an α of 0.05, a power of 0.8, a moderate effect size of (f) 0.28 [calculated based on the results from a previous study^29^, from mean (SD) variable error values of individuals with CLBP and asymptomatic controls during a multidirectional isometric tracking task at 90° and at 20–40% of maximal voluntary contraction (MVC)], and a potential 5% loss of data due to signal quality or participant withdrawal.

### Inclusion and exclusion criteria

Inclusion criteria for the CLBP group were men or women aged 18–55 years old, experiencing non-specific CLBP for a minimum of 3 months over the last 6-months^30^. Inclusion criteria for the asymptomatic control group were men or women aged 18–55 years old, without current or previous symptoms of back and/or lower limb pain which warranted attention from a health care professional. Due to the higher prevalence of age-related changes in the musculoskeletal system with increasing age, the upper age limit was set at 55, as applied previously^31^. The exclusion criteria for both groups were as follows: lumbar radiculopathy and/or radicular pain, spinal deformity and/or surgery, pregnancy, history of cardiovascular diseases, history of neurological and/or chronic respiratory problems and systemic or inflammatory conditions, including rheumatic and neuromuscular disorders. People with CLBP that sought treatment for their low back pain in the last 6 months before enrolment were also excluded.

### Questionnaires

At the beginning of the session, the 11-point Numerical Pain Rating Scale (NPRS) was verbally administered to all participants to obtain information about their current level of pain intensity. All participants were asked to indicate the numeric value that best described their pain intensity on a scale from 0 to 10, with 0 = “no pain” and 10 = “worst possible pain”^32^. All participants were also asked to report if they experienced any increase in their level of pain intensity after performing each of the contractions. The Oswestry Disability Index (ODI) was used to evaluate all participants’ perceived level of back disability during everyday activities of daily living^33^. All participants’ general health status was assessed with the RAND (research and development)-36-Item Health Survey^34^ and fear-avoidance beliefs and fear-avoidance behavior was assessed with the Tampa Scale for Kinesiophobia (TSK)^35^.

### Electromyography

**Figure 1:**
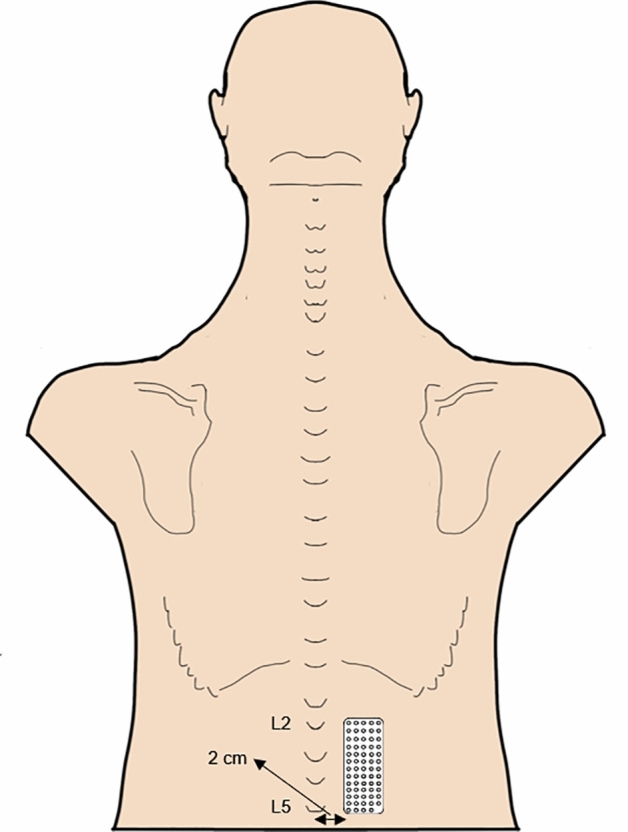
Representation of the position of the HDsEMG electrode grid that was placed over participants’ lumbar ES.

Surface HDsEMG signals were recorded in monopolar mode from the lumbar ES unilaterally, using a 13-row and 5-column semi-disposable grid of equally spaced electrodes (OT Bioelettronica, Italy). One electrode was missing from the right bottom corner of the grid, to provide directional reference^27^. Thus, each grid consisted of 64 electrodes of 1-mm diameter that were 8-mm apart. Prior to electrode placement, the HDsEMG grid was prepared by attaching a double-side adhesive foam to the electrode surface (SPES Medica, Genoa, Italy) and by filling the electrode cavities with a highly conductive-adhesive paste which provided electrode–skin contact (AC-CREAM, SPES Medica, Genoa, Italy). Participants’ skin preparation procedures were also performed prior to electrode placement, including shaving (if necessary), gentle local skin abrasion and cleaning with water. Then, the HDsEMG grid was placed unilaterally over the lumbar ES, 2 cm lateral to the L5 spinous process (AS controls: right side, CLBP group: most painful side) (Fig. 1)^23,24,26,36^. The electrode was placed on the most painful side for the CLBP group as it was expected that this side would show greater changes in myoelectric activity^25^. The portions of the ES which are likely to be muscular in the region of the electrode grid include the iliocostalis lumborum pars lumborum and the iliocostalis lumborum pars thoracis^27^.

One pair of bipolar electrodes (WhiteSensor WS, Ambu A/S, Ballerup, Denmark; total size: 36 × 40 mm, electrode size: 4 mm, centre-to-centre distance ≈3.6 cm) was mounted over the rectus abdominis (RA) muscle as described previously^37^ in order to be able to quantify the level of co-activation between the trunk flexors and extensors. The bipolar electrodes were placed on the same side as the HDsEMG grid, and the bipolar signals were recorded in differential mode. Ground electrodes (WhiteSensor WS, Ambu A/S, Ballerup, Denmark) were placed over the sacrum, anterior superior iliac spine (ASIS) and wrist of all participants.

The sEMG signals were synchronized with the torque signals acquired from the isokinetic dynamometer through the auxiliary input of the surface sEMG amplifier (Quattrocento- OT- Bioelettronica, Torino, Italy). All sEMG and torque signals were amplified by a factor of 150, sampled at 2048 Hz, automatically filtered with a band-pass filter (bandwidth: 10–500 Hz, first order, − 3 dB) and digitized with a 16-bit A/D converter.

### Dynamometer setup and positioning

The torque exerted by the participants during isometric MVCs and during isometric submaximal torque steadiness tasks was evaluated with an isokinetic dynamometer (System 3 Pro, Biodex Medical Systems, New York). All contractions were performed on the Biodex Dual Position Back Extension/Flexion Attachment.

**Figure 2:**
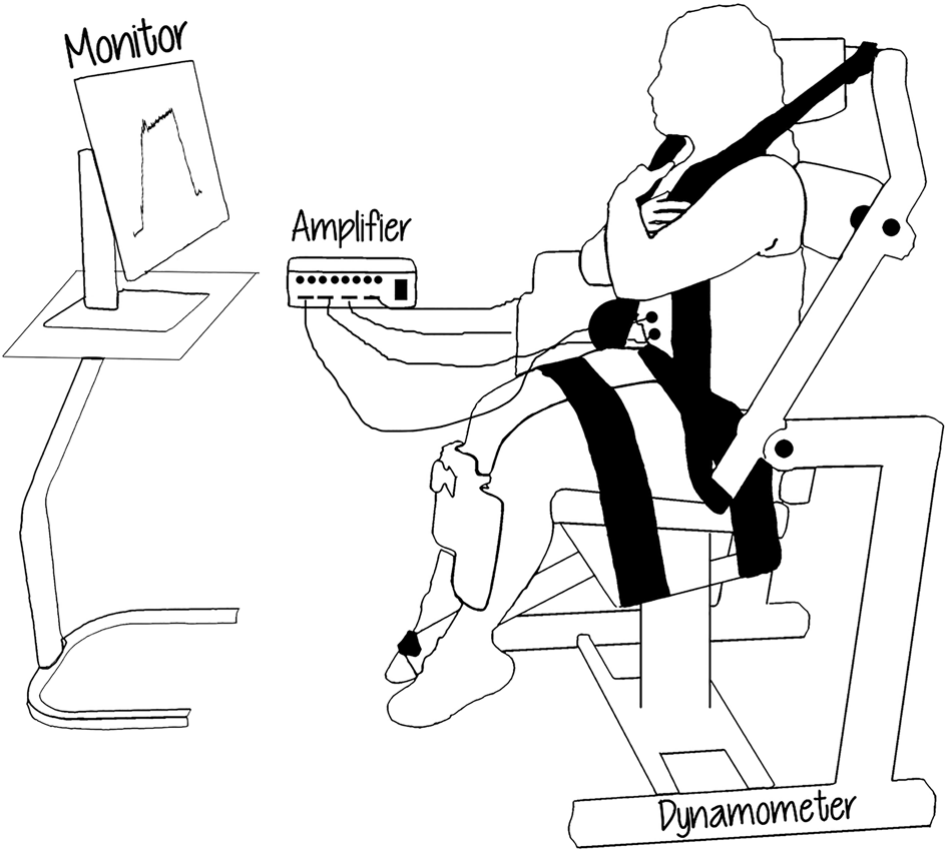
Representation of the testing procedure. A participant is seated on the Biodex chair with the electrodes attached to her and connected to the amplifier, and she performs isometric back extension at one of the two torque levels, while visual feedback of the torque output is provided to her via a monitor.

The participants were seated on the trunk attachment with their hips and knees in 90° of flexion, and their feet parallel to the floor at a distance equal to the distance of the two acromia (Fig. 2). The dynamometer was aligned bilaterally with the ASISs, while the front of the seat was tilted clockwise ≈15°. This position is referred to as “compressed isolated lumbar position” and was used to focus on the lumbar region^38^. In this position, the main contributor to trunk extension torque is the lumbar ES^39^. Additionally, the participants’ thighs, pelvis and upper trunk were strapped to minimize compensatory movements and a specific attachment was also used to block any knee movements.

For all participants and for both isometric back extension measurements (i.e., maximal, and sub-maximal), the dynamometer was locked with a hip-to-trunk angle of 90°. To achieve this hip-to-trunk angle, the dynamometer was locked at 50° according to the dynamometer’s goniometer for all participants.

### Testing protocol

After completing the questionnaires and electrode placement, the participants performed three submaximal isometric back extensions on the isokinetic dynamometer as a warm-up. After a brief rest, they were asked to perform three isometric back extension MVCs. Each of these trials lasted 5s and they were separated by 1 min of rest. The highest MVC value was used as a reference to define the submaximal torque level. After the MVC measurement, the participants rested for 5 min. Then, after completing a few practice trials, they were asked to perform one non-fatiguing sustained isometric back extension at 20% (2-s ramp-up and ramp-down contraction with 20s hold phase) and one at 50% (5-s ramp-up and ramp-down contraction with 15s hold phase) of the MVC, respectively. These submaximal torque levels were selected in order to replicate lumbar extensor contraction intensities utilised during activities of daily living^14^. The order of the contractions was randomized, and they were separated by a 2 min rest period. Real-time visual feedback of the trunk extensors torque output to the dynamometer, and a line indicating the target level of contraction (%MVC) was provided to all participants, via a computer monitor located 1.5 m in front of them (Fig. 2). During the submaximal contractions, the participants were first asked to match the %MVC target as accurately as possible, and once the requested target level was achieved (i.e., either 20 or 50%MVC), they were asked to try and maintain their torque as steady as possible for the full duration of the task. The instructions were given to all participants prior to the contractions. All participants were verbally motivated to exert their maximum torque during the MVCs. No verbal encouragement was provided during the two submaximal isometric lumbar extension tasks and the environment was kept silent.

### Torque signal analysis

The highest peak torque exerted during the MVCs (SI: Newton-meters) was used as a measure of maximal trunk extension strength for each participant. The absolute peak torque values were normalized to participants body mass (N⋅m⋅kg^−1^) to allow statistical comparisons between groups relative to body mass. The amplitude of the torque fluctuations was evaluated in both absolute and relative terms as the standard deviation (SD) of the torque signal and as the coefficient of variation (CoV) (CoV = SD/mean × 100) over time respectively. Additionally, the mean squared error (MSE) between the target and actual torque was computed to evaluate differences in torque accuracy. SD, CoV and MSE of torque were computed in the same time range used for the sEMG analysis during the two torque levels (i.e., steady part of the contraction). A custom-made MATLAB script was used to plot the torque exerted by each participant, visually identify the steady part of the contraction, and select the starting and ending point of the time window needed for our analysis.

### sEMG signal pre-processing

Prior to coherence and cross-correlation calculations, the 59 differential sEMG signals (formed by differentiating the 64 monopolar signals in the presumed direction of the muscle fibers) were processed as described previously^22^: (1) high-pass filtering (10 Hz) (PCA-input signals), (2) selection of a subset of channels by applying PCA to the 59 differential HDsEMG signals in the time domain (PCA-selected signals) (3) full-wave rectification of the selected channels and averaging them to get one time signal (Averaged PCA-selected signal), (4) low-pass filtering (10 Hz), which allows to identify slow frequency fluctuations in motor unit firing rate/recruitment^5^, (5) smoothing with a 1st order Savitzky-Golay filter and (6) DC (i.e., zero-frequency) components removal (Final Signal Envelope).

Due to the high dimensionality of the HDsEMG data, the recordings might contain redundant and correlated information (e.g. noise, artefacts and channels with low level of muscle activity) which challenges the interpretation of data^40^. An effective way of dealing with the high dimensionality of HDsEMG data is by using PCA. PCA is an unsupervised spatial filtering algorithm which aims at reducing the dimensionality of the data while at the same time retaining as much variance as possible, without losing any important information^41^. PCA has been used as a feature extraction/reduction technique for classification problems^21,22^. However, in more recent sEMG-related studies, it has been used as a pre-filter of highly correlated data for muscle estimation^21,22^. In our study we implemented PCA as a nonlinear dimensionality reduction technique over HDsEMG channels for each participant individually, to reduce a large set of correlated sEMG signals into a smaller subset of uncorrelated variables (principal components). In this way noise and redundant information is reduced and the dataset is simplified, allowing the identification of meaningful patterns. PCA decomposes the covariance structure of a set of dependent variables into orthogonal (uncorrelated) components by calculating the eigenvalues and eigenvectors of the data’s covariance matrix. These two algebraic formulations are always computed as a pair. Eigenvalues represent the percentage of the total variance that is informed by each eigenvector. Eigenvectors, also known as principal components (PC), represent the weight of each original dependent variable in explaining the portion of the variability defined by the eigenvalue^42^. PCA sorts the PCs in descending order of variances, meaning that as much activity as possible is accumulated in the first PCs^43^. Therefore, the first PCs are expected to contain most of the relevant information, and removing the remaining PCs, simply implies enhancing the signal-to-noise ratio^43^. According to Naik et al.^40^, it is reasonable to retain PCs that cover more than 80% of the data variance to reduce the dimensionality of the HDsEMG data. In the current study, the acceptable percentage of total variance was set to 85%. After organising the PCs in descending order of variance and maintaining those that cumulatively explained 85% of the variance, a new matrix with the PCs eigenvectors was generated. Notably, this was the first step towards dimensionality reduction, as by leaving out some PCs, the final dataset had less dimensions than the original (i.e., < 59). Given that in the previous steps no changes have been made to the original data, the last step of the PCA was to derive the new dataset (i.e., original data solely in terms of the vectors we chose). This was done, by using the PCs matrix formed by the eigenvectors of the covariance matrix to reorient the data from the original axes to the ones represented by the PCs. This was simply done by multiplying the transpose of the original dataset by the transpose of the PCs matrix. This led to a reduced dimensionality of the original dataset and allowed us to obtain a subset of HDsEMG channels.

After extracting the relevant force-related information (i.e., low frequency component) from the HDsEMG grid by applying PCA, coherence and cross-correlation analyses were conducted to quantify the degree of similarity between the Final Signal Envelope (i.e., the low pass filtered Averaged PCA-selected signal resulting from the first PCs that explained 85% of the variance, provided by the HDsEMG grid) and torque signals for each submaximal contraction. More specifically, as we also describe later in the cross-correlation and coherence analyses sections, the PCA-selected signals were averaged to generate one signal (Averaged PCA-selected signal). Then, the sEMG envelope obtained from this signal (Final Signal Envelope) was correlated with the torque signal in the time and frequency domains.

For the sEMG amplitude calculation, the monopolar HDsEMG signals were also differentiated in the presumed vertical direction of the muscle fibers (i.e., in the direction of the electrode columns) to form 59 adjacent bipolar channels, as mentioned earlier^24^. This resulted in 12 longitudinal bipolar channels in each column, except from the far right which had 11 electrode pairs (due to the missing electrode). Firstly, sEMG signals were bandpass filtered (10–350 Hz, second order, zero lag Butterworth)^25^. Then, bipolar channels with low signal-to-noise ratio due to poor skin–electrode contact or movement artifacts were removed after visual inspection of their quality. In the current study, the removal rate was < 15%.

### Cross-correlation analysis

To quantify the degree of correlation between fluctuations in sEMG and torque signals in the time domain, cross-correlation was used. More specifically, once the principal components which represented 85% of the variability were selected, the input data (PCA-input signals) was reoriented from its original axes to the principal components’ axes. In this way, a compressed representation of the HDsEMG signals was obtained which contained the maximum variance (PCA-selected signals). Then, the recast HDsEMG data obtained from PCA was averaged to get a single signal HDsEMG signal which explained most of the variance in sEMG activity (Averaged PCA-selected signal). The sEMG envelope of this signal (Final Signal Envelope), was then correlated with torque. Cross-correlation values can range between − 1 and 1 where 1 represents perfect correlation in phase and − 1 perfect correlation but in antiphase. Values around zero represent null correlation between signals. From the cross-correlation values the maximum peak was identified along with its time lag value.

### Coherence analysis

Coherence analysis was carried out to indirectly estimate the strength and frequency of common rhythmic synaptic inputs across the motor unit pool and establish their relationship with torque^44^. Coherence between sEMG and force signals were computed by employing magnitude squared coherence (MSC) with a 50% overlapping Hamming window of 1-s, as done previously^45–47^. Similarly to the cross-correlation analysis, the same sEMG envelope (obtained from PCA analysis; Final Signal Envelope) was then correlated with the torque signal in the frequency domain. The MSC approach has been previously used in many areas of signal processing^48^. It is a measure that estimates the extent to which one signal can be predicted from another signal using a linear model^48^. The MSC is a real number between zero and one at each frequency. If the MSC value is zero, this indicates that those signals are linearly independent at that frequency (no correlation), and if it is one, that they are perfectly correlated^49^. MSC calculations were performed by using the *mscohere* function of the Signal Processing Toolbox of MATLAB, which estimates MSC using Welch's overlapped averaged periodogram method (i.e., N sub-windows with 50% overlap). As described previously^50^, the MSC between two signals *x(t)* and *y(t)* can be computed as follows:

$$MSC_{xy} \left( f \right) = \frac{{\left| {P_{xy} \left( f \right)} \right|^{2} }}{{P_{xx} \left( f \right)P_{yy} \left( f \right)}}$$

where *P*_*xy*_*(f)* is the cross-power spectral density between the two time-domain signals *x(t)* and *y(t)*, and *P*_*xx*_*(f)* and *P*_*yy*_*(f)* are the auto-power spectral densities of *x(t)* and *y(t)* respectively. The magnitude of the spectral density is denoted as |P|. It should be also noted that cross-power spectral density represents the cross-correlation of two different signals on the frequency domain and auto-power density the correlation of one signal with itself^50^.

To enable statistical comparison of coherence values (C), they were converted to Fisher’s values (FZ) and the bias was then removed as shown in the equation below^51,52^. As it is possible that the sEMG recordings may be contaminated by some level of cross-talk, the coherence estimation may also be affected by this. Therefore, we applied the bias which is calculated empirically as the maximum value of coherence at 250 Hz, in which range is expected to find no correlation between the signals^47,51,52^.

$$FZ = \text{a}\tanh \left( {\sqrt C } \right) - bias$$

Since the Savitzky-Golay polynomial filter tends to preserve rapid fluctuations in sEMG signals^22^, we also analysed coherence at alpha (5–15 Hz) and beta bands (15–30 Hz). However, since none of these bandwidths showed significant coherence, we mainly focused on the analysis of the δ band (0–5 Hz) which is the most relevant for the generation of force^3^. The δ band coherence values were transformed to z-scores to allow statistical comparisons. Please note that the low frequency components < 5 Hz were filtered out during pre-processing, however, the subsequent full-wave rectification allows the analysis of the low-frequency modulation of the signal amplitude^53^.

Additionally, we created topographical maps of coherence (described below). In these maps, each of the 59 bipolar recordings was correlated with the torque signal in the frequency domain to provide one value. Then all the values were normalized to the maximum coherence value observed at each torque level respectively (i.e., 20% and 50%MVC). This map enabled the identification of areas of higher and lower coherence with torque, respectively. Finally, the centroid of coherence was calculated. This measure can provide an estimate of where the center of coherence is along the x- (medial–lateral) and y-axis (cranial-caudal). This allowed us to examine whether certain muscle regions contribute more to the exerted torque than others, by statistically comparing the x- and y-axis centroid values between groups.

### sEMG amplitude calculation

Root mean square (RMS) values were computed for each bipolar recording (i.e., 59 from ES and one from RA) and for each of the lumbar extension tasks (i.e., MVCs and two torque levels). The 59 RMS values were then averaged to form one value which was used as a global measure of the ES myoelectric activity (HDsEMG amplitude; ES RMS_mean_) during each task. Please note that this analysis was not the same as the one used for the coherence and cross-correlation analysis (i.e., rectification of the sEMG signal) mentioned earlier. RMS is commonly used to measure the level of muscle activation. Having one RMS value for each of the RA and ES muscles, enabled us to quantify co-activation (unilaterally) between the trunk flexors and trunk extensors during the isometric lumbar extension at the two different torque levels. The level of co-activation was calculated based on the non-normalised RMS values, as follows: antagonist muscle activity (RA RMS)/agonist muscle activity (ES RMSmean) *100. To allow between group comparisons, the ES RMS_mean_ values obtained during the two torque levels, were expressed as a percentage of the ES RMS value calculated during the maximal (baseline) MVC. The ES MVC RMS value was extracted separately for each participant. More specifically, from around the peak of the highest isometric MVC, 1 s of the contraction was cut and a non-overlapping 0.25 s sliding averaging window was used. In other words, one RMS value was produced each 0.25 s (i.e., 4 values in total) and then the average of those was used to calculate the max ES MVC RMS value.

Similarly, to others, all the aforementioned variables (except RMS value during MVC), were determined from the steady torque part of the contraction using a non-overlapping 0.5 s sliding averaging window (i.e., one average value was calculated for each variable, every 0.5 s)^54^. The length of the time windows used for the analysis was approximately 18 s and 13 s for the 20%MVC and 50%MVC torque levels respectively. This was performed to exclude the first and last second of the steady part of the contraction from our analysis, which were the time-instants that participants over or underestimated the requested torque level.

All HDsEMG and torque signals were analyzed offline, using a custom script on MATLAB 2020b (The MathWorks Inc., USA).

### Statistical analysis

All data analyses were performed on SPSS Statistics, version 27 (IBM, USA). The Shapiro–Wilk test was used to test the normality of the data and the assumption of homogeneity of variance was assessed with a Levene test. These assumptions were met, and thus parametric tests were applied. Descriptive statistics were used to analyze the data and the results are expressed as means and SDs. Anthropometrics, NPRs scores, questionnaire scores and isometric back extension MVC between groups were compared with independent *t*-tests. Statistical comparison for sEMG and torque variables between groups (RMS, Torque_mean_, CoV of torque, SD of torque, MSE, sEMG-torque coherence in the δ band, sEMG-torque cross-correlation, sEMG-torque coherence y- and x-axis centroid) was performed using a two-way mixed (i.e., with one within- and one between-subjects factor) analysis of variance (ANOVA), using torque target (20%MVC, 50%MVC) and group (CLBP, control) as within-subject and between-subject factors respectively. The level of co-activation between the ES and RA muscles was also evaluated with the same analysis. When significant interactions were revealed from the ANOVA, the Bonferroni method was used to allow pairwise comparisons. Pearson’s correlations evaluated associations between the sEMG parameters [variations (i.e., 20%MVC to 50%MVC change) in sEMG-torque coherence in the δ band, variations in sEMG-torque cross-correlation, variations in sEMG-torque coherence (δ band) y- and x-axis centroid and variations in the level of RA-ES co-activation and ES RMS amplitude)], the motor performance characteristics (variations in CoV and SD of torque) and the self-reported outcome measures (pain intensity, level of disability, general health and kinesiophobia). The level of statistical significance was set to *p* = 0.05.

## Results

### Participants

All 30 participants (15 CLBP, 15 controls) successfully completed the study, and their characteristics are reported in Table 1. The two groups did not differ in terms of demographic characteristics. However, as expected the individuals with CLBP presented with higher levels of disability and lower general health compared to the control group. The individuals with CLBP reported a current level of pain intensity of 2.5 (2.2) which is characterised as mild pain^55^. As anticipated, the controls did not report low back pain. None of the participants reported an increase in their level of pain intensity after performing the contractions.

**Table 1:** Characteristics of all participants separated by group.

Characteristic	Controls (15)	CLBP (15)	*p*-value
Gender (%males)	53.3	46.7	–
Age (years)	27.4 (4.9)	27.1 (9.3)	0.931
Height (cm)	171.3 (5.4)	169.9 (5.7)	0.501
Weight (kg)	73.7 (10.4)	69.3 (10.1)	0.264
BMI (kg/m^2^)	25.2 (3.5)	23.8 (2.7)	0.271
Average pain intensity (NPRS)*	0.0 (0.0)	2.5 (2.2)	< 0.001
ODI (%)*	0.0 (0.0)	14.9 (7.5)	< 0.001
RAND 36-item health survey*	89.6 (6.8)	64.1 (19.6)	< 0.001
TSK*	28.1 (6.0)	34.7 (5.2)	0.004
Isometric back extension MVC (N⋅m⋅kg^−1^)	3.7 (1.0)	3.2 (1.6)	0.258

Data are presented as mean ± SD. *CLBP* chronic low back pain. *BMI* body mass index. *NPRS* numerical pain rating scale. *ODI* Oswestry Disability Index. *RAND* Research and Development. *TSK* Tampa Scale for Kinesiophobia. *MVC* maximal voluntary contraction.

The asterisk (*) indicates statistically significant differences between groups.

### Torque

**Figure 3:**
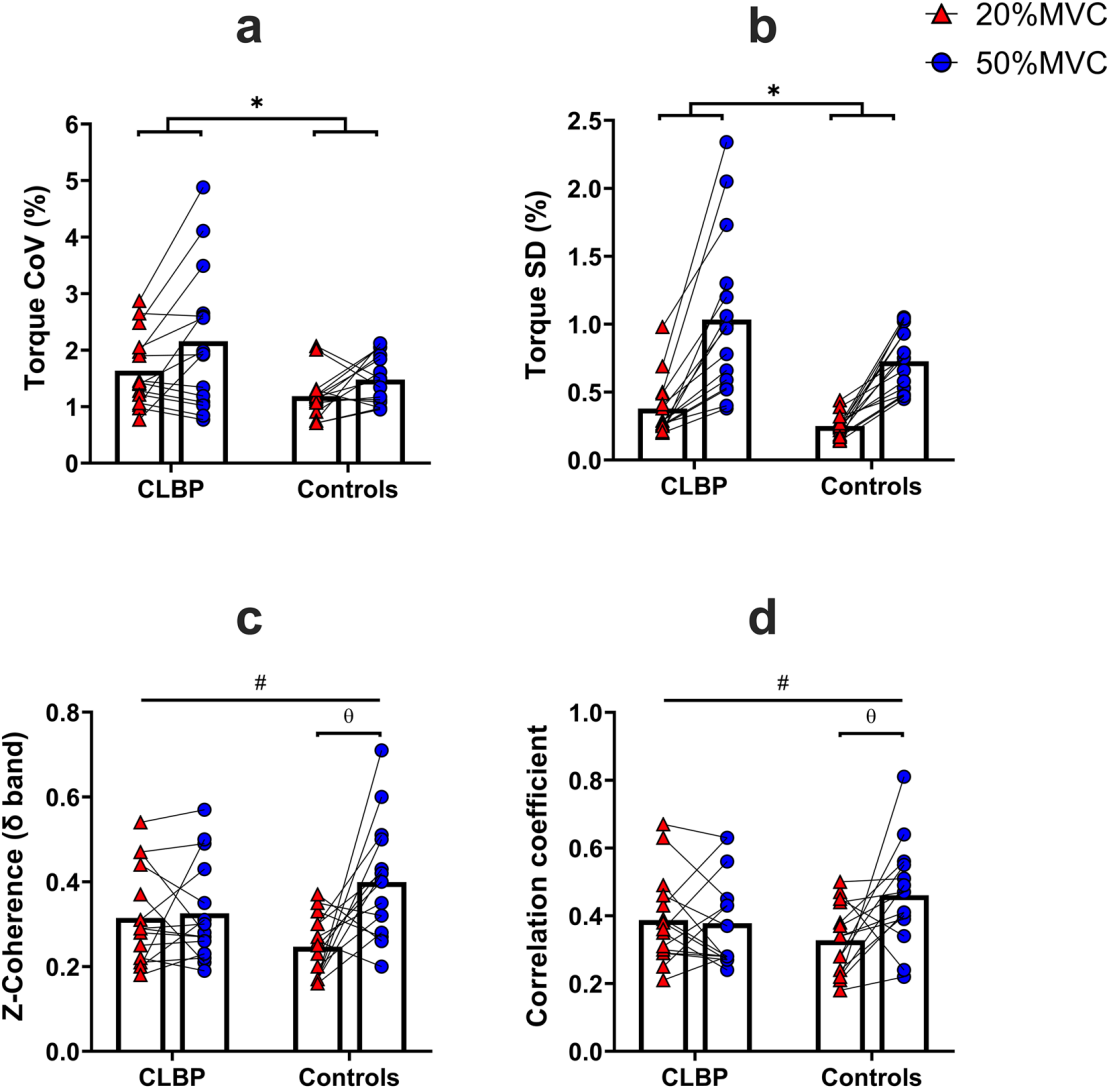
Torque CoV (**a**), torque SD (**b**), δ-band Z-coherence (i.e., δ band coherence values that have been transformed to z-scores) (**c**) and cross-correlation (**d**) values (mean ± SD) of the CLBP group and AS controls at 20% and 50%MVC respectively (*main effect of group, #interaction effect: group*torque, θ: post hoc tests).

Both groups exerted similar levels of isometric peak torque during the MVC of isometric back extension (Table 1). The mean submaximal torque exerted during the two torque levels was similar for both groups (F = 0.904, *p* = 0.350, ηp^2^ = 0.031). Overall, individuals with CLBP had significantly higher CoV and SD of torque than the asymptomatic controls during the two torque levels (main effect of group: F = 5.327, *p* = 0.029, ηp^2^ = 0.160 and F = 4.368, *p* = 0.046, ηp^2^ = 0.135 respectively; Fig. 3a,b). The MSE values were similar for both groups during the two torque levels (group effect: F = 3.670, *p* = 0.066, ηp^2^ = 0.116). The average MSE values for both groups and torque levels were as follows: Controls at 20%MVC and 50%MVC: 0.18(0.22) % and 1.44(1.15) % respectively, CLBP at 20% MVC and 50%MVC: 0.69(0.79) % and 2.43(2.06) % respectively.

### Electromyographic activity

The normalized spatial mean of the RMS values for the lumbar ES did not differ between groups during the two torque levels (group effect: F = 3.850, *p* = 0.060, ηp^2^ = 0.121). The mean values for both groups and torque levels were as follows, controls at 20%, 50%MVC: 37.8(19.9)% and 60.5(17.9)% respectively, CLBP at 20%, 50%MVC: 52.1(15.9)%, 76.1(29.9)% respectively. Both groups presented similar levels of RA-ES co-activation during the submaximal contractions (group effect: F = 0.010, *p* = 0.922, ηp^2^ =  < 0.001). The average RA-ES co-activation values for both groups and torque levels were as follows, controls at 20%, 50%MVC: 130.4(128.3)% and 84.9(62.5)% respectively, CLBP at 20%, 50%MVC: 114.9(89.9)%, 100.5(70.2)% respectively.

### Cross-correlation and coherence

The cross-correlation increased with the increase in torque for the asymptomatic controls, but this was not the case for the CLBP group (interaction: group × force, F = 5.234, *p* = 0.030, ηp^2^ = 0.157; Fig. 3d).

**Figure 4:**
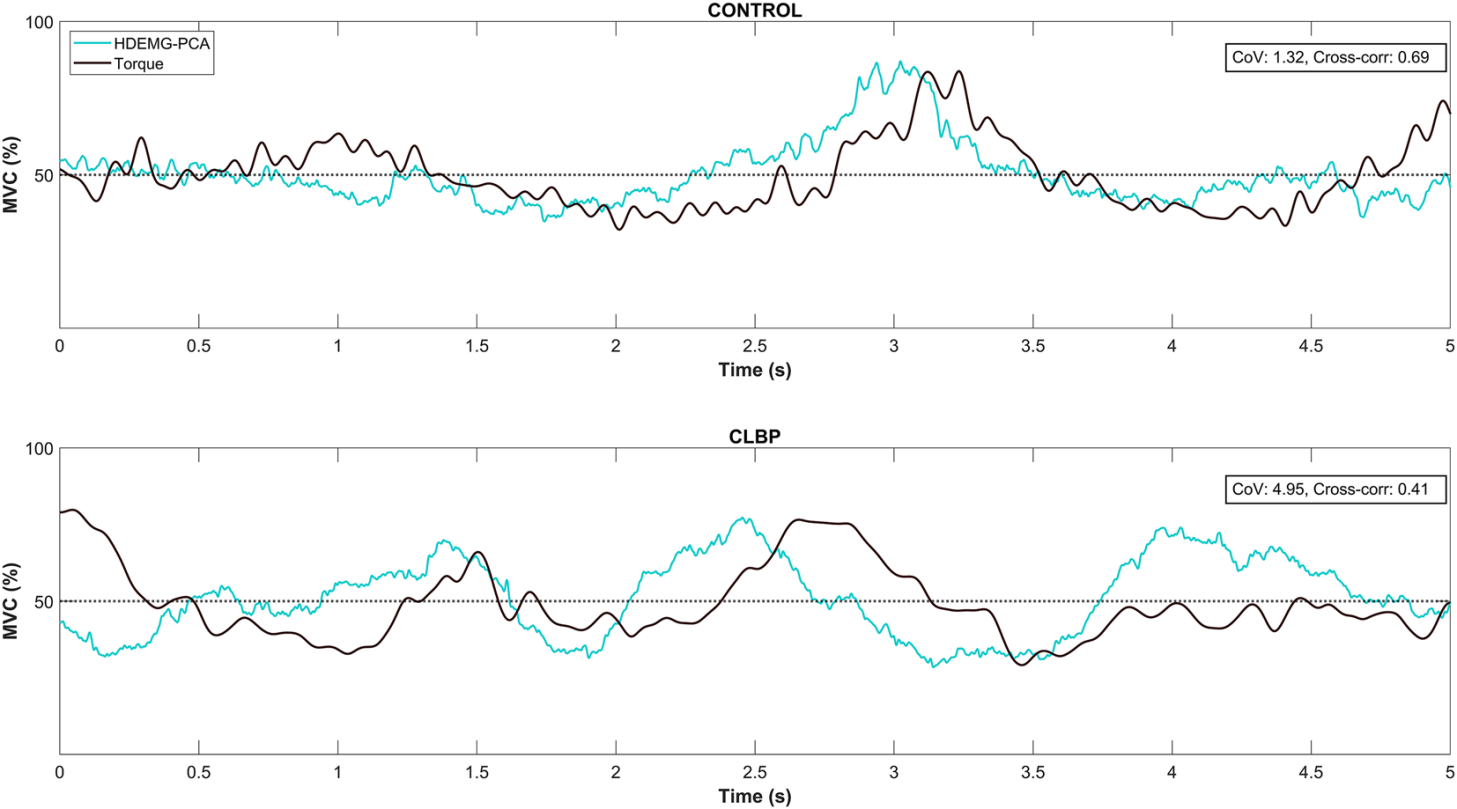
A sample outcome of lumbar extensor muscle torque steadiness assessment (5s window from the steady part of the contraction) for a control and a CLBP participant during the 50%MVC isometric back extension task. The black line represents the oscillations in torque for each participant, while the turquoise line represents the oscillations in the HDsEMG signal (i.e., the PCA-processed sEMG signal envelope). Note that the control participant has greater torque steadiness and at the same time a higher sEMG-torque cross-correlation, while the CLBP participant presents with lower torque steadiness performance and has a lower sEMG-torque cross-correlation at the same time.

**Figure 5:**
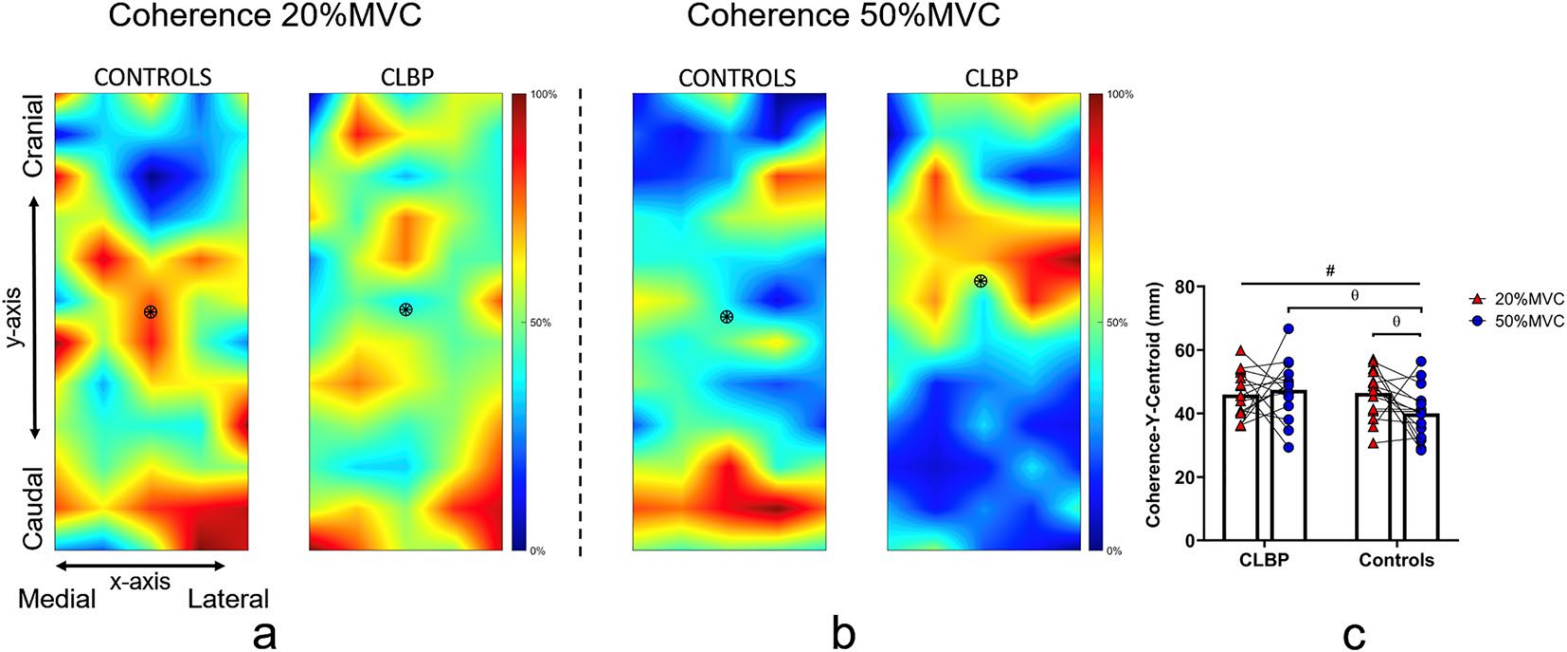
Topographical maps of averaged normalized (to the maximum coherence value) coherence across participants at (**a**) 20% and at (**b**) 50%MVC. The black circle in the topographical maps (**a**,**b**) represents the centroid of coherence, which was calculated from the bottom of the electrode grid. The spine is located towards the medial side of the x-axis. Figure (**c**) depicts the position of the y-axis centroid for both groups at 20% and 50%MVC (#interaction effect: group*torque, θ: post hoc tests). Hotter/red colors represent higher coherence, while blue/cold colors represent lower coherence.

Coherence in the δ band increased with the increase in torque for the asymptomatic controls but not in the CLBP group (interaction: group × force, F = 6.346, *p* = 0.018, ηp^2^ = 0.185; Fig. 3c). The centroid of coherence along the y-axis differed significantly between the two groups at 50%MVC, with higher coherence being observed in the more cranial region of the lumbar ES for the CLBP group (interaction: group × force, F = 4.511, *p* = 0.043, ηp^2^ = 0.139; Fig. 4). With the increase in torque, a higher coherence in the more caudal region of the lumbar ES was observed in the asymptomatic controls (interaction: group × force, F = 4.511, *p* = 0.043, ηp^2^ = 0.139; Fig. 5).

### Associations between outcome variables

No significant correlation (Pearson’s r) was observed between any of the sEMG, motor output (torque production) and self-reported (pain and function) parameters (*p* > 0.05 in all possible comparisons).

## Discussion

In this study we aimed to (1) measure the relationship between HDsEMG oscillations and isometric trunk-extension torque in both time and frequency domains, using cross-correlation and coherence analysis respectively, and (2) identify and compare regional differences in sEMG-torque coherence of the lumbar ES in individuals with CLBP and asymptomatic controls. sEMG-torque coherence in the δ band and sEMG-torque cross-correlation increased with the increase in torque for the asymptomatic controls, but this was not observed for the CLBP group. Additionally, the CLBP group had higher sEMG-torque coherence in more cranial regions of the lumbar ES at 50%MVC, while the opposite was observed for the asymptomatic controls (i.e., higher sEMG-torque coherence in more caudal regions of the lumbar ES). Interestingly, the level of lumbar ES activity (HDsEMG amplitude), and the co-activation levels did not differ between groups during the tasks.

### sEMG-torque coherence and cross-correlation alterations

We quantified the relative power only in the δ (0–5 Hz) frequency bandwidth from the interference sEMG and torque signal since torque fluctuations during isometric contractions are mainly explained by the effective neural drive to the muscle, i.e., low-frequency component (< 10 Hz) of the neural drive to the muscle^56^.

Interestingly, even though an increase in torque is commonly accompanied with an increase in δ band coherence^47^, as seen in the asymptomatic group, the CLBP group failed to increase sEMG-torque coherence with the increase in torque. This finding is also supported by the cross-correlation results, showing that in the CLBP group, the net increase in torque was not accompanied by an increase in the contribution of the lumbar ES muscle to the resultant torque fluctuations. Given that the low-frequency component of the rectified sEMG is associated with the variability in motor unit firing rate and hence torque oscillations^57^, correlation and coherence between sEMG and torque, should have been higher in the CLBP as they had higher variability in torque fluctuations compared to the asymptomatic controls. However, this was not observed, as at both torque levels, cross-correlation and coherence was similar for the CLBP group. Additionally, given that both groups exerted a similar amount of torque during the task, the individuals with CLBP could have compensated by activating synergistic muscles. Under the influence of pain, the central nervous system can find a new motor strategy to achieve a specific task (e.g., perform an MVC and/or an isometric torque steadiness task in our case) and due to the redundancy of the trunk motor control system, people with CLBP often display substantial reorganization of their motor control strategies^58^. This can allow people with CLBP to adopt different muscle recruitment solutions to achieve a specific outcome and possibly avoid pain. Importantly, these changes can be explained by alterations of the descending drive to the muscles, but also by changes in motor planning (i.e., modulated by cognitive/emotional factors)^59^. In the present study, individuals with CLBP had higher levels of Kinesiophobia (higher scores in TSK) compared to the asymptomatic controls. Thus, it is possible that the CLBP group experienced more fear as the torque increased, leading to the use of compensatory strategies, which likely involved the hip extensors, and this would explain why we did not observe an increase in the sEMG-torque coherence and cross-correlation values in the δ band (i.e., no increase in the common synaptic input and the contribution of the lumbar ES muscle to the net torque). This is also further supported by a previous observation, showing that initially pain-free individuals with high pain-related fear adopt avoidant strategies during common reaching movements shortly after the induction of delayed-onset muscle soreness^60^. However, it should be noted that this is speculative, more so considering that the average TSK score of 34.7 for the CLBP group is still considered low. Future studies could investigate this further. Interestingly, the levels of lumbar ES activation and ES-RA co-activation were similar between the two groups. This shows that RMS amplitude alone (i.e., using a simple estimate of muscle activation) could not explain any differences between the two groups in the execution of the torque steadiness task, but the proposed coherence analysis gave us better insight into the actual contribution of the trunk extensor muscles to the resultant torque.

### Regional variations in HDsEMG-torque coherence

Moreover, given that regional differences in myoelectric activity of lumbar ES is a common finding in people with CLBP^23–27,61^ and that altered distribution of input to the motoneurons can change motor unit recruitment patterns during pain^62^, we investigated whether regional differences in sEMG-torque coherence also existed between people with CLBP and asymptomatic controls. Differences in topographical representation of coherence were observed at 50%MVC, since the lumbar ES coherence centroid in the CLBP group was displaced more cranially (suggesting higher coherence in the upper region of the muscle), while in the asymptomatic controls, the centroid of coherence was displaced towards more middle-caudal regions of the lumbar ES. This finding likely suggests that that torque is less represented in the caudal region of the muscle in individuals with CLBP at 50%MVC, since the correlation between sEMG and torque is the lowest in this region. Since common synaptic input is reflected by δ band coherence, this could suggest that individuals with CLBP show reductions in sEMG-torque relationships due to a more focalized distribution of common synaptic input (HDsEMG-torque coherence) of the lumbar ES and possibly a higher influence of independent synaptic inputs on the lumbar ES motor unit pool, at least when they exert higher forces. However, these findings should be confirmed with sEMG-torque coherence, based on sEMG decomposition analysis, as the interference sEMG signal is influenced by several factors^19^. From a clinical perspective, it should be also noted that this more focalized contribution of muscle fibers to the resultant torque, imply that those specific muscles fibers (mainly located in the cranial region of ES) will be the main contributors to the resultant torque during extension. This could possibly lead to an uneven distribution of load, thus, overloading of those specific muscle fibers^61^. Such motor behaviour could possibly play an important role in the perpetuation of CLBP symptoms.

### Impaired torque control in people with CLBP

As anticipated, people with CLBP had reduced trunk extensor muscle torque steadiness, compared to asymptomatic controls (Fig. 4). These findings are in line with those from previous studies^14,15^ and further support the notion that the control of muscle force (measured either as force accuracy and/or force steadiness/variability) is impaired in individuals with CLBP. Trunk muscle function impairments are common in people with CLBP, and these can be more pronounced when motor control is challenged by tasks that require high precision, such as the one used in the current study^15^. These impairments, including reduced torque steadiness, are likely attributed to the numerous functional and structural neuroplastic changes that can occur at multiple levels of the nervous system (i.e., periphery, spinal and/or supraspinal centers) when people have pain^9^. For example, proprioceptive acuity is reduced in people with CLBP^9^, and this can be related to suppression of proprioceptive input by nociceptive afference and/or altered afferent input due to changes in the mechanical properties of passive structures (e.g., ligaments and intervertebral discs)^7,8^. Additionally, it could be related to structural changes, such as the reduction of white matter integrity of the superior cerebellar peduncle, which is an important brain area for the transmission of proprioceptive input to higher centers^63^. Importantly, previous studies have also shown that regions of the motor cortex that are related with the control of specific trunk muscles are also reorganized in people with CLBP (overlap between muscle cortical representations), and this is likely related to a loss of discrete control of paraspinal muscle fascicles^64,65^. Collectively, these neuroplastic changes observed in people with CLBP could explain their impaired capacity to control trunk muscle force accurately. Compensations for proprioceptive deficits and/or other neuroplastic changes can be reflected as alterations in the efferent motor response (i.e., motor recruitment strategy). Therefore, it is relevant to unravel the motor control strategy that the CLBP group utilized to exert the requested amount of torque during the torque steadiness and MVC tasks.

### Trunk muscle strength

Trunk extensor isometric muscle strength did not differ between groups, which is consistent with the results of some previous studies^14,23,66^. Previous research has shown that pain decreases the ability to exert maximal forces^67,68^, due to nociceptive-induced inhibition to agonist muscles^69^. However, these findings are mainly observed in upper or lower-limb muscles, which produce torque around a single joint (i.e., tibialis anterior)^67^ or across two joints (i.e., biceps bracchii)^68^. On the contrary, trunk extension is a multi-joint movement that involves concurrent movement of the lumbar spine, pelvis and hips^70^. Therefore, people with CLBP could have performed the MVC by compensating with the large hip extensor muscles (e.g., gluteus maximus and hamstrings), which can influence both the hip and pelvis, thereby minimizing the activation of their lumbar extensors. Although, the thighs and pelvis were stabilised with straps, it is not possible to completely prevent such compensatory strategies. These factors could have allowed the participants to perform the MVC with a higher contribution of their hip extensors, compared to their lumbar extensors (possibly due to pain and/or fear). As previously shown, when the thigh is stabilised, the hamstrings and the gluteus maximus rotate the pelvis posteriorly which likely “disengages” and counteracts lumbar extension^71^. Thus, during the MVC, people with CLBP could have exerted torque by extending their hips with their pelvis tilted posteriorly (i.e., with higher contribution of the gluteal and hamstring muscles). It could be also argued that people with CLBP managed to exert a similar amount of torque during the MVC by greater activation of deeper lumbar extensor muscles, such as the multifidus muscle, however, this is unlikely as this muscle’s main function is to stabilize the lumbar spine and is more commonly inhibited in people with CLBP^72^.

### Lack of associations between outcome variables

Lastly, it should be noted that there was no significant correlation between the sEMG parameters, the motor output (torque production) parameters and the self-reported measures of pain and function. This makes it difficult to explain the observed changes in the sEMG parameters and means that the neuromuscular mechanisms responsible for the poorer trunk muscle torque steadiness observed in individuals with CLBP, warrant further investigation. However, this is not a surprising finding, as many studies do not report any significant correlation between motor output and self-reported measures of pain/function^73–75^ and/or sEMG parameters^15,76^ in people with musculoskeletal pain. Some possible reasons for the lack of correlation between these parameters are the following: (1) the fact that people with CLBP present a wide variety of neuromuscular responses and that there is substantial variability even considering one type of neuromuscular adaptation, (2) the characteristics of the individuals with CLBP recruited for this study (i.e., mild symptoms), and (3) the variability in the questionnaire data due to their subjective nature. It is likely that each participant with CLBP used a different strategy to maintain a steady force during the task regardless of the level of pain and/or changes in function^77^. However, this should be confirmed in future studies which incorporate recordings from multiple muscles during similar tasks in order to obtain greater insight of the potential compensatory strategies utilized by people with CLBP.

### Methodological considerations and limitations

A limitation of this study is that the sample consisted mainly of young, active individuals with low pain intensity and mild disability. This likely limits the generalisability of the results. However, even with relatively low levels of disability and pain intensity we were still able to identify impairments in this group, although it should be noted that there was variability between individuals with CLBP, as has been commonly observed previously^24^. It is likely that a sample of people with more severe CLBP would present with even higher deficits, or other neuromuscular adaptations which could negatively impact on the ability to control torque output in a steady way. Additionally, this study did not measure possible compensatory patterns by using kinematic markers and/or recording sEMG activity of synergist muscles. Another limitation that should be considered is that the rectified interreference sEMG is only a rough estimator of the neural drive to the muscles, as it is influenced by several factors, including amplitude cancellation (i.e., the overlapping of motor unit action potentials)^78^. Based on the findings of a simulation study, amplitude cancellation can distort the spectrum of the rectified sEMG signal and thus have an influence on the results^78^. This problem is commonly solved by basing the analysis on the identification of motor unit spike trains (i.e., by using sEMG decomposition), rather than on sEMG. However, HDsEMG decomposition is currently challenging on signals collected from the lumbar ES likely due to factors such as the (1) complexity of lumbar anatomy (i.e., existence of many different layers of muscles and thoracolumbar fascia)^79^, and (2) the volume conductor properties of the lower lumbar area (i.e., thicker subcutaneous layer)^20^. These factors can reduce the discriminative information in the action potential waveforms of different motor units, and thus limit the applicability of decomposition algorithms^20^. Therefore, we based our analysis on the rectified sEMG signal. Nevertheless, the correlation between the sEMG signal (i.e., PCA-processed sEMG signal envelope) and torque was ≈ 0.4 for both groups and torque levels, likely suggesting a moderate correlation between sEMG and torque oscillations. Studies employing motor unit recordings usually report higher discharge rate vs torque cross-correlation values (approximately 0.75–0.80)^5^. However, this is usually seen in smaller, single-joint muscles (i.e., tibialis anterior). It is likely that these correlations would be reduced in larger muscles like ES, however, this is yet to be determined. Lastly, another limitation that needs to be addressed is the calculation of co-activation based on non-normalised RA and ES RMS values, which might have provided less reliable co-activation estimates due to inter-subject anatomical differences (e.g., differences in sub-cutaneous tissue thickness between the abdominal and paraspinal regions). Nevertheless, in our previous study, co-activation was calculated based on normalised RA and ES RMS values during a dynamic isokinetic fatiguing task and again we did not observe differences in co-activation between people with CLBP and asymptomatic controls^23^. On top of this, whether absolute or normalised sEMG-RMS amplitudes are favorable in the interpretation of co-activation index remains unclear^80^. Thus, we believe that similar findings would have been observed, even if the RMS values were normalised during the calculation of co-activation ratios.

## Conclusion

This study uniquely demonstrates that individuals with CLBP fail to increase the common fluctuations in torque and HDsEMG activity, when exerting higher lumbar extension forces. This reduced ability to alter the sEMG-torque relationship, is likely due to regional adjustments of ES-sEMG oscillatory activity (torque was not represented in the caudal region of the lumbar ES in individuals with CLBP) and the use of compensatory trunk extension patterns during this task. The current study contributes to the existing body of knowledge and further supports the notion that individuals with CLBP have poorer control of their trunk extensor muscles compared to asymptomatic individuals.

## Ancknowledgements

We would like to acknowledge the contribution of Bikinis Nikolaos, Petrakis Stylianos, Gkioka Afroditi and Tsimpolis Dimitrios, students at the School of Sport, Exercise and Rehabilitation Sciences of the University of Birmingham, for their help with the experimental procedure. We would also like to thank the volunteers participating in this study.
